# Development of a Virtual Transition Program for Adolescents and Young Adults With Type 1 Diabetes: Three-Stage User-Centered Design Study

**DOI:** 10.2196/91543

**Published:** 2026-07-16

**Authors:** Sarah Cary Haynes, Patrick S Romano, Salvador Lopez, Erin Conboy Heiser, Allison Reggiardo, Bethany M Kwan, Elaine H Morrato, Jennifer L Rosenthal, Miriam Sarkisian, Abigail Leon, Deborah Plante, Stephanie S Crossen

**Affiliations:** 1 Department of Pediatrics, UC Davis Health, 4610 X Street, Suite 2300, Sacramento, CA, 95817, United States, 1 9167345627; 2 Center for Healthcare Policy and Research, UC Davis Health, Sacramento, CA, United States; 3 Center for Health and Technology, UC Davis Health, Sacramento, CA, United States; 4 Clinical and Translational Science Center, UC Davis Health, Sacramento, CA, United States; 5 Department of Medicine, UC Davis Health, Sacramento, CA, United States; 6 Department of Emergency Medicine, University of Colorado School of Medicine, Aurora, CO, United States; 7 Parkinson School of Health Sciences and Public Health, Loyola University Chicago, Chicago, IL, United States

**Keywords:** type 1 diabetes, adolescents, human-centered design, transition to adult care, peer support

## Abstract

**Background:**

Adolescents and young adults (AYAs) with type 1 diabetes (T1D) face a heightened risk of care gaps and preventable complications during the transition from pediatric to adult care. AYAs from low-income families are more likely to experience poor outcomes. Care guidelines for T1D now recommend a transition program beginning several years prior to anticipated independence. However, existing transition programs often require substantial resources, limiting their scalability and accessibility.

**Objective:**

We used a human-centered design approach to develop and refine a low-cost, virtual intervention to support T1D transitions among publicly insured AYAs.

**Methods:**

Guided by the “3I” model (inspiration, ideation, implementation) of IDEO, we conducted semistructured interviews with 26 providers and staff across 4 University of California medical centers to identify challenges and opportunities for improving transition care. We then completed customer discovery interviews with 36 adopters and influencers, including AYAs, endocrinologists, and diabetes educators, to identify priorities and needs and create value propositions. The resulting intervention was tested with 3 cohorts (n=25) of publicly insured AYAs aged 17 to 21 years receiving pediatric diabetes care at an academic medical center. The intervention consisted of two 75-minute virtual group sessions led by a diabetes educator and peer mentors with lived experience. Feasibility, acceptability, and appropriateness were assessed via validated surveys and qualitative interviews.

**Results:**

Core functions of the intervention included education and discussion on “adulting with diabetes” and navigating the health care system, interactive creation of a transition plan, mentors sharing their experiences with diabetes, open discussion among peers, opportunities to ask questions, and provision of resources based on individual needs. Facilitators and mentors reported high feasibility (mean 4.25, SD 0.52) and appropriateness (mean 4.43, SD 0.53). Qualitative interviews highlighted peer support and lived-experience mentorship as core drivers of engagement. Attendance was highest among participants enrolled closer to session dates. Participant feedback informed ongoing adaptations to content and delivery across cohorts, including timing of the intervention, use of the chat function, multiple text reminders before intervention sessions, and specific topics of interest.

**Conclusions:**

This human-centered virtual transition intervention addressed priorities identified by AYAs, peer mentors, and clinicians, including peer support, judgment-free discussion, and self-advocacy. While engagement and attendance varied across cohorts, findings support further refinement and evaluation of the intervention in diverse settings.

## Introduction

Type 1 diabetes (T1D) is a serious medical condition that affects more than 300,000 children and adolescents in the United States [1]. T1D poses a significant burden on patients and families in terms of health-related quality of life, missed school days, missed work days, and health care costs [2-4]. Previous research suggests that increased independence and a desire for autonomy during adolescence and young adulthood may lead to reduced adherence to medications and other self-care strategies, as well as lapses in communication with health care providers [5-9]. The transition from adolescence to young adulthood has been identified as a particularly vulnerable stage for lapses in care; up to one-third of patients with T1D fail to complete an initial visit with an adult endocrinology provider within 12 months after their final pediatric endocrinology visit [10,11]. For patients with T1D, the transition phase is associated with high rates of hospitalization due to diabetic ketoacidosis (DKA), a life-threatening complication that can be prevented with appropriate management [9,12]. Patients from low-income families are the most vulnerable to these lapses in care and subsequent hospitalizations; intervening in the transition period is therefore essential to prevent the widening of disparities into adulthood [12,13].

Current guidelines from the American Diabetes Association suggest that the transition process from pediatric to adult specialty diabetes care should begin at least 1 to 2 years prior to the actual transfer of care to allow for a gradual increase in adolescents’ self-management responsibilities and continuous education on navigating the logistical aspects of adult health care [14]. Previous T1D transition programs have demonstrated success in improving continuity of care and reducing hospitalizations using full-time transition coordinators, case management, designated young adult clinics, delaying transition until age 25 or 26 years, and shared medical appointments for adolescents and young adults (AYAs) [15-19]. However, more recent studies highlight ongoing gaps in transition preparation, including unmet psychosocial needs and variability in both transition readiness and the effectiveness of existing interventions [20-22]. In addition, transition interventions have not been systematically implemented, even at large academic medical centers, due to limited resources. We sought to design a low-resource, sustainable T1D transition intervention that could be easily implemented and scaled with existing resources within pediatric diabetes clinics. Here, we describe the human-centered design (HCD) process based on the “3I” framework of IDEO used to develop and test the transition intervention, as well as the key components we identified as essential to transition programs for AYAs with T1D.

## Methods

### HCD and Intervention Development Process

We used an HCD approach based on the “3I” model of IDEO, which includes 3 phases: inspiration, ideation, and implementation [23,24]. Across the 3 phases, we engaged distinct participant groups and data collection approaches, including provider and staff interviews (phase 1), stakeholder customer discovery interviews (phase 2), and intervention delivery with surveys and qualitative interviews among AYAs and mentors (phase 3). We incorporated the principles of HCD throughout, including engaging stakeholders early and throughout the design process, understanding the needs of patients, clinicians, and staff involved in diabetes care, and taking a systems approach to design [25,26]. We enhanced standard HCD steps with rigorous qualitative and customer discovery methods [27], adapted for implementation science [28], to aid in a rigorous, adopter-centered assessment of the “jobs, pains, and gains” that would be addressed by the transition intervention. Collectively, this iterative process followed best practices for designing for dissemination and sustainability and ensuring strong fit-to-context [29].

### Phase 1: Inspiration

In the first phase of the project, we gathered perceptions and ideas from providers and staff involved in delivering specialty diabetes care across 4 University of California medical centers. An open-ended question about transitions from pediatric to adult care was added to a semistructured interview guide for an existing qualitative study examining telehealth use for specialty diabetes care. This study used purposive and snowball sampling to identify adult and pediatric endocrinologists, nurses, and staff involved in specialty diabetes care. Interviews lasted 1 hour, were conducted on Zoom, and were recorded and transcribed. We conducted thematic analysis using line-by-line coding in Dedoose (Sociocultural Research Consultants, LLC) [30], as fully described elsewhere [31]. Responses to the transition question were coded and analyzed separately using qualitative content analysis. The research team organized and discussed the main findings for this question.

### Phase 2: Ideation

The second design phase was customer discovery, a process widely used across industries to identify intervention adopters’ and influencers’ “jobs, pains, and gains” and to develop value propositions tailored to different types of stakeholders (ie, adopters and influencers) involved in the clinical transition workflow [32-34]. Our process, adapted for implementation science, focused on understanding (1) adopters’ and influencers’ perspectives on the most important jobs to be done regarding transitions for patients with T1D, (2) the “pains” (ie, current challenges in supporting transition) and “gains” (ie, opportunities for improved experience or impact) for each group, and (3) how each group defines success [27,28,35]. We first developed a value proposition hypothesis, then conducted customer discovery to validate this hypothesis, and finally established a final value proposition statement. Value proposition statements articulate how the proposed intervention will relieve pains and create gains while carrying out the jobs to be done. Four groups of adopters and influencers were included in customer discovery: (1) people with T1D who had experienced the transition from pediatric to adult care in the past 10 years, (2) pediatric endocrinologists, (3) adult endocrinologists, and (4) diabetes educators. We recruited participants through a snowball sampling strategy, beginning with the participants from the phase 1 qualitative study. Customer discovery interviews were conducted over the telephone and lasted between 15 and 30 minutes; the interview guide was informed by standard customer discovery questions [27]. Questions for people with diabetes included the following: (1) Can you tell me about your experience transitioning from pediatric to adult care? (2) What types of things were you most concerned about? (3) What were the main challenges and how did you deal with them? (4) Is there anything that could have made the transition easier? (5) What would an ideal transition intervention look like? (6) How would you define a successful transition? Questions for providers and staff included the following: (1) What is your role in transitions of care for patients with T1D? (2) Can you walk me through a typical transition? (3) How do you feel your health system/department/division/clinic is doing at transitions? (4) What resources are offered? (5) How do you think transitions could be improved? (6) How important do you think transitions are (for you, your colleagues, your patients, and leadership)? (7) What motivates you to want to improve transitions? Responses were captured in notes during interviews and were summarized immediately afterward.

### Phase 3: Implementation

#### Participants

To refine the intervention components identified in phases 1 and 2, we tested our intervention with 3 cohorts of 6 to 10 AYAs. Participants were eligible for the study if they were (1) currently receiving pediatric endocrinology care at UC Davis, (2) between the ages of 17 and 21 at the time of enrollment, (3) planning to transition to adult endocrinology care at UC Davis, (4) insured under Medicaid or California Children’s Services (CCS) insurance, which we used as a proxy for low-income status, and (5) able to participate in English-language video sessions. Participants were recruited by a trained clinical research coordinator both during in-person visits and through messages sent via the electronic health record (EHR). The clinical research coordinator confirmed the eligibility criteria and obtained informed consent as described below.

#### Intervention and Adaptations

The first of our 3 cohorts received three 75-minute group virtual sessions led by a diabetes nurse educator and supported by 2 “T1D mentors,” adults aged 23 to 35 with T1D who had previously transitioned to adult endocrinology care. Initial content included modules on the following: (1) navigating the health care system, (2) “adulting” with diabetes (eg, alcohol, pregnancy, social interactions, and explaining diabetes to others), and (3) developing a transition plan. Sessions were conducted synchronously via Zoom and included a combination of facilitator-led discussion, mentor storytelling, and interactive activities. At the end of each session, we asked for feedback from participants on the intervention components, discussion topics, and meeting format. Feedback was tracked and discussed with the research team to make subsequent adaptations to sessions. Two subsequent cohorts of AYAs received the adapted intervention.

#### Qualitative Interviews

Following the completion of each cohort, we invited all participants and T1D mentors to participate in a qualitative interview about their experiences with the intervention. Interviews were completed via Zoom or phone, lasted approximately 30 minutes, followed a semistructured interview guide, and were audio-recorded and transcribed. Deidentified transcripts were coded line-by-line by 2 independent coders. Coders met regularly to review codes and resolve discrepancies through discussion. Final themes were discussed, categorized, and refined by the research team. Findings from interviews from the first 2 cohorts were also incorporated into intervention adaptations.

#### Survey Measures

All participants completed a baseline survey on demographics, employment, educational status, and time since T1D diagnosis. After completing the intervention, facilitators and T1D mentors completed 2 short validated surveys that measure perceived intervention appropriateness and feasibility on a 5-point Likert scale: the intervention appropriateness measure and the feasibility of intervention measure [36]. Participants were also asked to complete a third validated measure, the acceptability of intervention measure. Surveys were analyzed using means and SDs.

### Ethical Considerations

This study was reviewed and approved by the UC Davis Institutional Review Board (approval number 1982341). Interview participants for phases 1 and 2 provided verbal consent. AYA participants in phase 3 provided written informed consent if aged 18 years or older and written assent along with written informed consent from the parent or guardian if younger. Participants who turned 18 during the study were reconsented.

## Results

### Phase 1: Inspiration

Qualitative study participants included 5 pediatric endocrinologists, 7 adult endocrinologists, 4 diabetes educators, 2 diabetes pharmacists, and 8 staff members involved in specialty diabetes care. We identified 3 main findings related to transitions from pediatric to adult care for persons with T1D: (1) There is a perception among all types of clinicians and staff that transitions to adult care need improvement; (2) Levels of support for transitions vary widely depending on the patient’s distance from the health system; and (3) Components that may be helpful for a transition program include navigating the health care system independently (eg, insurance, appointments, pharmacy, and device companies) and a transition plan available to all members of the care team (pediatric and adult) in the EHR.

### Phase 2: Ideation

We completed customer discovery interviews with 36 participants, including 16 people with diabetes, 8 pediatric endocrinologists, 6 adult endocrinologists, and 6 diabetes educators. Adults with diabetes who had previously transitioned emphasized that, during that period of their lives, their primary concern was not health outcomes per se but succeeding in broader developmental milestones, such as starting college, entering the workforce, or living independently. They described the transition as most effective when it supported their ability to manage diabetes in a way that enabled these life goals, rather than focusing narrowly on avoiding adverse health events like DKA or hospitalization. When reflecting on what would have helped them most during the transition, participants consistently identified 3 essential components: access to peer support, a space to ask questions without judgment, and the development of strong self-advocacy skills. Table 1 summarizes the motivating gains, pains, and value proposition statements for each adopter or influencer group.

**Table 1.** Motivating “pains and gains” and value propositions by adopter or influencer group.

Group	Motivating “gains”	Current “pains”	Value proposition statement
AYA^a^ with T1D^b^	More independence and confidence as they move into a new phase of life (eg, entering the workforce, attending college, living independently).	Not knowing others with T1D diabetes (“no one to talk to about it”), anxiety about navigating the health system, anxiety about asking for accommodations.	For adolescents and young adults with T1D, this intervention provides all of the information they need to transition to adult care in one place along with a supportive, judgment-free environment where they can learn from diabetes experts and peers, they can call on for help in the years to come, allowing them to focus on living their life instead of on their diabetes.
Pediatric endocrinologists	Greater ability to provide resources to ensure a smooth transition to adult care.	Emotional pain seeing patients not successfully transition, discomfort around continued insulin prescriptions after leaving pediatrics.	For pediatric endocrinologists who want to ensure the patients they have cared for for so long end up thriving after transition, this intervention provides them with an easy way to increase that likelihood and feel good about the resources being provided to their AYA patients.
Adult endocrinologists	Greater confidence that AYAs are arriving to adult care with more education and connection to resources.	High no-show rates for AYAs, which contribute to inefficient use of physician and staff time and may lead to poor outcomes for patients.	For adult endocrinologists who want to avoid poor clinical outcomes and reduce no-shows for new AYA patients, this intervention provides an easy way to help these patients transition to their care and gives them the opportunity to take advantage of the wealth of knowledge gathered on these patients over the years by their pediatric colleagues.
Diabetes educators	Having a more convenient resource available for all AYAs.	Limited time to provide individualized education and transition resources to every patient.	For diabetes educators that want to provide as much support as possible to all of their AYA patients, this intervention allows them to do this in a convenient way that will give them the opportunity to participate as much as they would like but won’t place a mandatory burden on their time.

a AYA: adolescents and young adult.

b T1D: type 1 diabetes.

Across interviews with pediatric endocrinologists, adult endocrinologists, and diabetes educators, several key themes emerged about the transition from pediatric to adult diabetes care. Pediatric endocrinologists saw their primary role as initiating transition conversations and referring patients to adult care, often with support from diabetes educators. Many described significant emotional distress when patients failed to transition successfully, especially when long-term patients experienced poor outcomes. They also expressed discomfort about continuing to refill insulin prescriptions for patients who should have moved on, and they described time and resource limitations as ongoing challenges. Adult endocrinologists focused more on ensuring that young adults received the care and tools they needed to manage diabetes in adulthood. While they reported less emotional strain, given they typically meet patients after the transition, they highlighted high no-show rates and limited background information as key pain points. Diabetes educators emphasized their role in providing resources and answering questions about what to expect in adult care but noted that strict oversight of how they use their time limited their ability to support transitions more fully. They were enthusiastic about anything that could reduce the burden of 1:1 education and help more patients. Customer discovery significantly influenced the intervention design, including prompting the addition of “T1D mentors,” adults who have transitioned in the past 10 years, to share their experiences and strategies, and having a specific focus on advocating for oneself (eg, explaining diabetes to others, asking for accommodations, etc). These features created the “gains” sought by participants in terms of connecting with other people with T1D and improving confidence, and addressed the “pains” of anxiety around self-advocacy.

### Phase 3: Implementation

We identified 73 eligible patients at the beginning of the study. Of these, 25 were enrolled through in-clinic recruitment and EHR messaging. Seven eligible participants declined, citing that they were either not interested in research (n=4), had a work conflict (n=1), were concerned about the time commitment (n=1), or did not feel comfortable participating in English (n=1). The remaining 41 did not respond to StudyPages (Yuzu Labs PBC) messages and were not seen in the clinic during the recruitment window. Table 2 shows the demographic and health characteristics of participants for phase 3 testing. The group was diverse, with 48% (12/25) female participants, 40% (10/25) of Hispanic or Latino ethnicity, and a variety of self-identified races. The median age of participants was 18 (IQR 18-19), and median number of years living with diabetes was 6 (IQR 4-12.5). Self-reported employment status varied, with 44% (11/25) enrolled as students (including 3/25, 12% with part-time jobs), 12% (3/25) working part-time, 4% (1/25) working full-time, and 20% (5/25) unemployed and not in school.

**Table 2.** Characteristics of participants in phase 3 user testing.

Characteristics	Values (N=25)
Sex, n (%)	
Male	13 (52)
Female	12 (48)
Race, n (%)	
White	9 (36)
Black	6 (24)
Asian	3 (12)
Native Hawaiian or Pacific Islander	1 (4)
Other	6 (24)
Ethnicity, n (%)	
Hispanic or Latino	10 (40)
Not Hispanic or Latino	15 (60)
Employment, n (%)	
Student only	8 (32)
Student also employed part-time	3 (12)
Part-time job	3 (12)
Full-time job	1 (4)
Unemployed	5 (20)
Missing	5 (20)
Age, median (IQR)	18 (18-19)
Years living with type 1 diabetes, median (IQR)	6 (4-12.5)

The intervention for the first cohort consisted of three 75-minute sessions scheduled monthly. In the first cohort, 7 out of 9 participants enrolled attended the first session, but only 3 participants attended the subsequent 2 sessions, and only 2 participants completed all 3 sessions. We subsequently adapted the intervention to 2 sessions delivered over consecutive weeks in response to feedback from cohort 1 participants. In the second cohort, 5 out of 8 participants enrolled attended session 1, and 4 also attended session 2. In the third cohort, only 2 out of 8 participants enrolled attended the first session, and only 1 attended both sessions. Attendance appeared to be influenced in part by the timing of the sessions relative to enrollment, with lower participation observed when there was a longer delay between enrollment and the session start.

After the completion of cohort 1, we also increased the use of the chat function in subsequent sessions, as many participants were uncomfortable speaking on video but actively participated in the chat when asked to do so. We also adapted the transition plan component of the intervention; rather than asking participants to fill out and share their transition plans, participants preferred to watch one of the T1D mentors fill out the transition plan template and discuss the rationale behind their choices. Table 3 shows the resulting core components and adaptive forms of the intervention.

**Table 3.** Core functions of the transition intervention, refined over 3 stages of human-centered design.

Core functions of transition intervention	Components
Education and discussion on “adulting with diabetes” facilitated by a certified diabetes educator	Asking for accommodations at work or school Advocating for oneself Talking about diabetes with others Understanding the relationships between diabetes and alcohol, drugs, sleep, stress, pregnancy, and driving Managing diabetes while away from home Being prepared for emergencies
Education and discussion on navigating the health care system as an independent adult with T1D^a^	Making appointments Asking diabetes-related questions and finding information Filling prescriptions Implications of insurance changes Communicating with device companies Obtaining additional supplies and replacement devices Expectations for adult care
Interactive creation of a detailed transition plan	Provide fillable online template Talk through examples as a group
“T1D mentors” sharing experiences with T1D	Between 2 and 4 T1D mentors during each session provide stories and differing perspectives about how they navigated the transition to adult care
Open discussion between peers with T1D	Provide time between topics and at the end of sessions for open discussion
Opportunities to ask any questions about T1D management to peers, T1D mentors, and facilitators	Encouragement to discuss T1D mentors facilitate discussion and questions
Assess individual needs and provide additional resources as needed	Facilitator and T1D mentors provide links to informational resources based on group discussion or questions Participants were encouraged to use chat during session or to email afterwards with questions

a T1D: type 1 diabetes.

We completed a total of 15 qualitative interviews (9 participants, 5 T1D mentors, and 1 facilitator) during phase 3. Table 4 summarizes qualitative findings and provides illustrative quotes. Participants described the intervention as helpful, prompting them to think about aspects of managing their care they had not previously considered, and improving their confidence that they would transition successfully to adult care. The 2 components highlighted by participants as being most valuable were the T1D mentors and peer support. Many participants had not previously spoken with other people with T1D and appreciated the open discussions about challenges others had encountered and how they overcame them. These findings suggest that the core needs identified during customer discovery, particularly peer support, judgment-free discussion, and self-advocacy, were reflected in participants’ experiences of the intervention. T1D mentors and facilitators also perceived the intervention to be valuable. Mentors and participants had different perspectives on session participation. While mentors were concerned that some participants were not engaged in the sessions, participants themselves noted that, although they may have had their cameras turned off or not spoken often, they were nonetheless engaged and learned from the discussions between the peer mentors and other participants. Qualitative interviews also provided insights into the intervention timing, ideal number of participants, and perspectives on topics and exercises.

**Table 4.** Key findings from qualitative interviews with adolescent and young adult (AYA) participants and type 1 diabetes (T1D) mentors and facilitators.

Finding	Illustrative quote	Implications for transition intervention
AYAs found interaction with peers with T1D to be supportive and encouraging; peer support may mitigate some of the barriers to transitioning successfully (eg, feeling discouraged or lonely).	“I think hearing from other people my age and what they deal with really helped me realize I’m not alone. We’ve all got to deal with it, and we are still trying to accomplish everything even though we have diabetes.” [AYA participant]	Highlight this aspect of the intervention in recruitment and during sessions. Encourage further peer-to-peer support to reduce isolation and foster motivation.
AYAs found the discussion with T1D mentors, who shared their experiences managing T1D, how they overcame challenges, and strategies they use to manage health, social situations, and logistics, to be extremely valuable.	“At first, I was kind of scared because I didn’t know how I was going to do everything on my own. But now I feel like I am better. I see other people who have done this and I kind of think to myself, if they can do it, I can too.” [AYA participant]	Incorporate structured opportunities for mentors to share experiences and strategies (eg, structured questions, preparation in advance of sessions).
Personal stories from the T1D mentors can help make AYA participants feel more comfortable in the group setting.	“Yeah, I definitely felt comfortable because the fact that everybody had the same thing as I do and especially people sharing, like, how [one mentor] talked about how her mom was kind of micromanaging and really on her about her numbers and all that. I can relate to that. It made me kind of come forward and speak about my own experiences.” [AYA participant]	Include mentor storytelling to normalize challenges and encourage peer discussion.
Navigating the health care system, including insurance, device companies, and pharmacies, will present challenges for many AYAs. Discussing prior to independence or transition may be helpful.	“Just knowing where to get your stuff, like prescription and stuff, knowing where your closest pharmacy is. I didn’t even think about that because I’m going to be in charge and stuff like that now too. I might be doing things with friends and stuff, and this is all kind of going to be different.” [AYA participant]	Ensure that practical content on health care navigation is covered.
The transition intervention may increase self-efficacy regarding transition.	“I’ve been nervous about transitioning to an adult endocrinologist. And I found it very helpful that it gave me the peace of mind that it would be easy to transition.” [AYA participant]	Emphasize skill-building and reassurance to boost confidence in managing transition.
Some participants may be uncomfortable being on camera but want to participate in the discussion.	“Personally, I didn’t turn on my camera because I feel like I can still stay engaged without it on. And it makes me more awkward, I guess.” [AYA participant]	Allow flexible participation modes (eg, camera optional) to support comfort and engagement.
Icebreakers at the beginning of the session were helpful for improving comfort and engagement.	“I liked how they asked little questions, like what is your favorite low blood sugar snack…it gave me a feeling of like we all are dealing with the same thing and made it more comfortable to open up.” [AYA participant]	Incorporate icebreakers consistently to build connection and lower barriers to participation.
Participants found that a variety of different media and activities (eg, a mix of icebreaker questions, presentation by the facilitator, structured questions, and unstructured discussion time) were helpful for staying engaged.	“I thought the slide show was great…because the information was really helpful and stuff that we need to know about. And then you could also ask questions about other things if you wanted to.” [AYA participant]	Use diverse formats to maintain engagement and accommodate different learning styles.
T1D mentors were enthusiastic about sharing their experiences to help young people with T1D.	“I enjoyed it so much that I would love to do something like that again…so I think it would interest a lot of others like me to do it because it’s not often that you get an opportunity to be a mentor to somebody who is experiencing something you’ve already experienced.” [T1D Mentor]	Build sustainability by recruiting and supporting mentors who find value in contributing.

Seven facilitators and mentors (100% response rate) completed the intervention appropriateness measure and feasibility of intervention measure surveys. They rated the intervention as having good feasibility (mean 4.25, SD 0.52) and appropriateness (mean 4.43, SD 0.53). Of the 14 AYAs who participated in the sessions, only 3 completed the acceptability of intervention measure survey. These 3 participants rated the intervention as having high acceptability (mean 4.67, SD 0.29).

## Discussion

This study describes the development and iterative refinement of a human-centered virtual transition intervention designed to support AYAs with T1D during the transition from pediatric to adult care. The intervention was intentionally designed to align with existing clinic workflows and resources while addressing priorities identified by AYAs, peer mentors, and clinicians. Stakeholder input was gathered at every stage, including from patients, peer mentors, pediatric and adult providers, and educators. Across phases, we identified peer support, judgment-free education, and the development of self-advocacy skills as essential components of an effective transition program. Through iterative fit-to-context validation and refinement, we arrived at a virtual, mentor-supported intervention focused on practical concerns such as navigating insurance, managing diabetes in educational or work settings, and advocating for accommodations. While these components were consistently identified as valuable by participants, this did not necessarily translate into sustained participation, underscoring the distinction between intervention content and participant engagement.

Consistent with previous studies, participants described the transition as a time of vulnerability, not only because of elevated risk for adverse health events but also due to major life changes such as moving out, starting college, or entering the workforce [37-39]. While DKA and hospitalizations are often used as clinical indicators of a failed transition, participants in this study defined success differently. For them, successful transition meant maintaining diabetes care in ways that supported broader developmental goals. These findings highlight the importance of aligning intervention content with the real-world priorities of young adults rather than focusing solely on clinical end points [40].

Our intervention shared several elements with prior programs, including peer mentorship and a focus on building self-management skills [16,17,41-43]. However, unlike many existing models that depend on intensive resources, such as full-time coordinators or dedicated young adult clinics [44], this intervention was designed for use in settings with limited capacity. The structure of 2 virtual sessions, led by a diabetes educator and supported by trained mentors, may offer a practical approach for clinics with limited resources to support transition care. This is especially relevant for clinics serving large numbers of publicly insured patients or those with limited infrastructure to support transition care. However, efficiency is dependent on participant engagement, and low attendance may limit cost-effectiveness and scalability in practice. Prior educational and transition-focused interventions for AYAs with T1D have similarly reported mixed findings regarding engagement and retention, with some programs demonstrating strong participation and others encountering substantial challenges with sustained involvement [16,45-47]. Difficulty engaging AYAs and families in structured diabetes education has also been identified as an important implementation barrier, even in larger, well-resourced programs [48].

We also identified several important findings related to implementation. We observed lower attendance in the third cohort, which may be partially explained by the longer delay between enrollment and the start of the sessions. More broadly, engagement and retention were variable across cohorts, reflecting common challenges in delivering interventions during the transition stage. Future programs may benefit from enrolling participants closer to the intervention start date or developing strategies to maintain engagement during waiting periods. In addition, small adaptations, such as increased use of the chat function and a shift toward observational learning, were well received by participants. These changes reflect the value of responsiveness to participant preferences and highlight how flexible delivery can enhance engagement. Future adaptations may explore shorter or more flexible session formats to better align with participant preferences and competing demands.

A key strength of this study is its focus on publicly insured AYAs, a group often underrepresented in transition research and at increased risk for poor transition outcomes. AYAs from low-income households may face additional barriers, such as limited access to technology, competing work responsibilities, or less involvement from parents or caregivers. These factors can affect both participation in and the success of structured transition programs. Future research should explore how to further reduce these barriers. Potential strategies include integrating content into clinical workflows, using mobile-first platforms, or offering incentives for completion. This study has several limitations. First, the phase 3 testing was conducted at a single academic medical center in Northern California, which may limit generalizability. While some features of the intervention may support adaptation in other underserved settings (eg, the virtual format, limited number of sessions, and use of existing clinic personnel), implementation and engagement may differ in settings with fewer institutional resources, different staffing models, rural populations, or more limited access to reliable technology. Second, the phase 3 testing experienced notable attrition across sessions, with declining attendance over time. Interpretation of feasibility, appropriateness, and acceptability findings is therefore limited by low participant survey response rates and variable attendance. In addition, a substantial proportion of eligible participants were not reached or did not respond to recruitment, underscoring the challenge of engaging AYAs during the transition stage. These individuals may represent those at highest risk for care gaps, suggesting a need for alternative outreach and engagement strategies. Finally, the study focused on user testing and did not assess clinical outcomes, such as appointment attendance or hospitalization rates. Future work should evaluate the impact of this intervention on health outcomes and explore adaptation for other populations, including those in rural areas or non–English-speaking groups.

## Abbreviations

AYA: adolescents and young adults
CCS: California Children’s Services
DKA: diabetic ketoacidosis
EHR: electronic health record
HCD: human-centered design
T1D: type 1 diabetes

## References

[1] (2024). National diabetes statistics report. https://usdss.cdc.gov/diabetes/report.html.

[2] Sussman M, Benner J, Haller MJ, Rewers M, Griffiths R (2020). Estimated lifetime economic burden of type 1 diabetes. Diabetes Technol Ther.

[3] Milton B, Holland P, Whitehead M (2006). The social and economic consequences of childhood-onset type 1 diabetes mellitus across the lifecourse: a systematic review. Diabet Med.

[4] Kalyva E, Malakonaki E, Eiser C, Mamoulakis D (2011). Health-related quality of life (HRQoL) of children with type 1 diabetes mellitus (T1DM): self and parental perceptions. Pediatr Diabetes.

[5] Houtrow AJ, Newacheck PW (2008). Understanding transition issues: asthma as an example. J Pediatr.

[6] Withers AL, Green R (2019). Transition for adolescents and young adults with asthma. Front Pediatr.

[7] Lotstein DS, Seid M, Klingensmith G (2013). Transition from pediatric to adult care for youth diagnosed with type 1 diabetes in adolescence. Pediatrics.

[8] Sheehan AM, While AE, Coyne I (2015). The experiences and impact of transition from child to adult healthcare services for young people with type 1 diabetes: a systematic review. Diabet Med.

[9] American Diabetes Association Professional Practice Committee (2022). 14. Children and adolescents: standards of medical care in diabetes-2022. Diabetes Care.

[10] Daneman D, Nakhla M (2011). Moving on: transition of teens with type 1 diabetes to adult care. Diabetes Spectr.

[11] Pacaud D, Yale JF, Stephure D, Trussell R, Davies HD (2005). Problems in transition from pediatric care to adult care for individuals with diabetes. Can J Diabetes.

[12] Fingar KR, Reid LR (2021). Diabetes-related inpatient stays, 2018. https://www.ncbi.nlm.nih.gov/books/NBK573775/pdf/Bookshelf_NBK573775.pdf.

[13] Agarwal S, Kanapka LG, Raymond JK (2020). Racial-ethnic inequity in young adults with type 1 diabetes. J Clin Endocrinol Metab.

[14] ElSayed NA, McCoy RG, Aleppo G (2025). 14. Children and adolescents: standards of care in diabetes-2025. Diabetes Care.

[15] Spaic T, Mahon JL, Hramiak I (2013). Multicentre randomized controlled trial of structured transition on diabetes care management compared to standard diabetes care in adolescents and young adults with type 1 diabetes (Transition Trial). BMC Pediatr.

[16] Sequeira PA, Pyatak EA, Weigensberg MJ (2015). Let’s empower and prepare (LEAP): evaluation of a structured transition program for young adults with type 1 diabetes. Diabetes Care.

[17] Butalia S, Crawford SG, McGuire KA, Dyjur DK, Mercer JR, Pacaud D (2021). Improved transition to adult care in youth with type 1 diabetes: a pragmatic clinical trial. Diabetologia.

[18] Farrell K, Griffiths R, Fernandez R (2014). Factors determining diabetes care outcomes in patients with type 1 diabetes after transition from pediatric to adult health care: a systematic review. JBI Database Syst Rev Implement Rep.

[19] Buschur EO, Glick B, Kamboj MK (2017). Transition of care for patients with type 1 diabetes mellitus from pediatric to adult health care systems. Transl Pediatr.

[20] Cudizio L, James S, Maruthur NM (2025). Transition between pediatric and adult diabetes healthcare services: an online global survey of experiences and perceptions of young people with diabetes and their carers. Horm Res Paediatr.

[21] Papadakis JL, DeLacey S, Snoek FJ (2025). A systematic review of factors associated with transition readiness and the transfer to adult health care for young people with diabetes. Clin Diabetes.

[22] DeLacey S, Papadakis J, James S (2025). A systematic review of interventions for the transition to adult healthcare for young people with diabetes. Curr Diab Rep.

[23] Tschimmel K Design thinking as an effective toolkit for innovation. https://www.researchgate.net/publication/236135862_Design_Thinking_as_an_effective_Toolkit_for_Innovation.

[24] Zamakhsyari F, Fatwanto A (2023). A systematic literature review of design thinking approach for user interface design. JOIV Int J Inform Vis.

[25] Göttgens I, Oertelt-Prigione S (2021). The application of human-centered design approaches in health research and innovation: a narrative review of current practices. JMIR mHealth uHealth.

[26] Melles M, Albayrak A, Goossens R (2021). Innovating health care: key characteristics of human-centered design. Int J Qual Health Care.

[27] Osterwalder A, Pigneur Y, Bernarda G, Smith A (2015). Value Proposition Design: How to Create Products and Services Customers Want.

[28] Morrato EH, Bloom M, Thomas M, Rivera M, Mora N (2026). Building implementation science capacity: adaptation of I-Corps™@NCATS Training for rapid fit-to-context discovery and designing for scale-up and sustainability. J Clin Transl Sci.

[29] Kwan BM, Brownson RC, Glasgow RE, Morrato EH, Luke DA (2022). Designing for dissemination and sustainability to promote equitable impacts on health. Annu Rev Public Health.

[30] Dedoose.

[31] Haynes SC, Sarkisian M, Neinstein AB (2024). From surviving to thriving: a qualitative study of adapting telehealth systems for specialty diabetes care across four California medical centers. Clin Diabetes.

[32] Oh A, Gaysynsky A, Knott CL, Nock NL, Erwin DO, Vinson CA (2019). Customer discovery as a tool for moving behavioral interventions into the marketplace: insights from the NCI SPRINT program. Transl Behav Med.

[33] Thamjamrassri P, Song Y, Tak J, Kang H, Kong HJ, Hong J (2018). Customer discovery as the first essential step for successful health information technology system development. Healthc Inform Res.

[34] Concannon TW, Meissner P, Grunbaum JA (2012). A new taxonomy for stakeholder engagement in patient-centered outcomes research. J Gen Intern Med.

[35] Mora N, Mehall M, Lennox LA, Pincus HA, Charron D, Morrato EH (2024). A national unmet needs assessment for CTSA-affiliated electronic health record data networks: a customer discovery approach. J Clin Transl Sci.

[36] Weiner BJ, Lewis CC, Stanick C (2017). Psychometric assessment of three newly developed implementation outcome measures. Implement Sci.

[37] Vitale RJ, Toschi E, Ortega J (2025). Life stage transitions for people with type 1 diabetes. Lancet Diabetes Endocrinol.

[38] Garvey KC, Beste MG, Luff D, Atakov-Castillo A, Wolpert HA, Ritholz MD (2014). Experiences of health care transition voiced by young adults with type 1 diabetes: a qualitative study. Adolesc Health Med Ther.

[39] Hislop AL, Fegan PG, Schlaeppi MJ, Duck M, Yeap BB (2008). Prevalence and associations of psychological distress in young adults with type 1 diabetes. Diabet Med.

[40] Leung JMWS, Tang TS, Lim CE, Laffel LM, Amed S (2021). The four I’s of adolescent transition in type 1 diabetes care: a qualitative study. Diabet Med.

[41] Carreon SA, Minard CG, Lyons SK (2024). DiaBetter Together: clinical trial protocol for a strengths-based peer mentor intervention for young adults with type 1 diabetes transitioning to adult care. Contemp Clin Trials.

[42] O’Hara MC, Hynes L, O’Donnell M (2017). A systematic review of interventions to improve outcomes for young adults with type 1 diabetes. Diabet Med.

[43] Mackett K, Greenway M, Sherifali D (2026). Implementation of a peer mentorship program for type 1 diabetes in Canada: a qualitative analysis of peer mentor perspectives. Patient Educ Couns.

[44] Schultz AT, Smaldone A (2017). Components of interventions that improve transitions to adult care for adolescents with type 1 diabetes. J Adolesc Health.

[45] Harris S, Miller A, Amiel S, Mulnier H (2019). Characterization of adults with type 1 diabetes not attending self-management education courses: the barriers to uptake of type 1 diabetes education (BUD1E) study. Qual Health Res.

[46] Temple B, Epp D (2009). Evaluation of a diabetes education program’s non-attendees: the program response. Can J Diabetes.

[47] Caccavale LJ, LaRose JG, Mazzeo SE, Bean MK (2023). Development and implementation of a pilot transition preparation intervention for young adults with type 1 diabetes in an integrated healthcare setting. J Pediatr Psychol.

[48] Hagger V, Hendrieckx C, Cotterill A, Speight J (2026). Evaluation of a structured type 1 diabetes education program for adolescents and parents: Teens Empowered to Actively Manage Type 1 (TEAM T1). Diabet Med.

